# Treatment of Diphtheria

**Published:** 1891-02

**Authors:** 


### Treatment of Diphtheria.

That this disease is to be classified among the acute infectious disorders, dependent upon a specific infectious micro-organism, is now generally believed. Whether the disease is at first a local trouble with secondary infection of the organism, or whether it is a constitutional affection from the beginning, with local manifestations, are questions upon which the profession is still divided. Certain it is that abundant clinical experience has taught the value of antiseptic applications to the affected part. This rule has been evolved in the face of constant reiteration of the constitutional nature of the infection, and the consequent valuelessness of such local treatment. We may remark in this connection that recent experimental work upon the Klebs-Loffler bacillus has shown that it is capable of producing an exceedingly poisonous albuminoid, when grown in nutrient media. This experimental fact lends additional weight to the clinical value of local antiseptic measures in this disease.

Dr. Herman Wolf, *Therapeutische Monatshefte*, September, 1890) has borne strong testimony regarding the antiseptic value of methol in these cases. He proceeds according to the following method: A powder is prepared containing one part to ten or twenty of sugar, this, by means of a camel's hair pencil, is carefully applied to the false membrane and inflamed mucosa, which should have been previously cleansed from all secretions; if the nose is involved small quantities are blown into the anterior nares and post-pharyngeal space. If the process has extended to the bronchai, it may be employed in the form of a fine spray by inhalation. He claims for methol that it is quite free from toxic properties, is pleasant in odor and taste, and has a greater antiseptic value than most of the usual gargles and sprays. Of the value of complete and early local antisepsis he has no doubt.—*Journal of Amer. Med. Asso.*

THE University of Basel, the only university in Switzerland that has excluded women students, has just decided to admit them to the medical department.

